# Non‐A to E hepatitis in children: Detecting a novel viral epidemic during the COVID‐19 pandemic

**DOI:** 10.1111/1751-7915.14329

**Published:** 2023-08-21

**Authors:** Harald Brüssow

**Affiliations:** ^1^ Laboratory of Gene Technology, Department of Biosystems KU Leuven Leuven Belgium

## Abstract

During the COVID‐19 pandemic, two further novel viral epidemics were described in 2022, monkeypox virus infections in men having sex with men and non‐A to E hepatitis in children. The latter occurred in the first half of 2022 with about 1000 cases worldwide, necessitating liver transplantation in 5% and causing death in 2% of patients. It took some effort to clarify the cause of the novel hepatitis epidemic. Researchers were confronted with a polymicrobial viral infection consisting of an adenovirus‐associated virus type 2 (AAV2) infection, co‐occurring with either human adenovirus type 41 (HAdV41) or herpesvirus infections; most prominently human herpesvirus type 6 (HHV‐6). AAV‐2, a small Dependovirus of the Parvovirus family, needs these helper viruses for its replication. AAV2 is used as a vector for liver‐targeting gene therapy but was not previously known to cause acute hepatitis. HAdV41 and HHV‐6 are mostly known to cause diarrhoea and febrile illnesses associated with skin rashes in children, respectively. Except for a few case reports of HHV‐6 hepatitis, HAdV and HHV‐6 are mostly known as major pathogens in immunosuppressed transplantation patients. A potential role of SARS‐CoV‐2 has also been discussed but the most popular hypothesis involves an indirect role of the COVID‐19 pandemic for this novel disease. Exposure to HHV‐6 infections occurs nearly quantitatively during the first year of life. Social distancing measures, followed by the lifting of these measures in 2022 might have caused a delayed exposure to multiple, normally benign childhood viral infections eliciting a dysregulated immune response with pathological effects for liver cells. In the fall of 2022, when these conditions were not longer met, case numbers dwindled. The hypothesis of an unequilibrated immune response instead of intrinsic cytopathic activity of the implicated viruses is further supported by the enrichment of a particular HLA allele in cases over controls.

Pandemic preparedness has many aspects that range from early warning systems of pending viral threats at the animal–human interface, over the early detection of clusters of new viral infections in the human population, to designing early public health measures to restrict the further spread of new infections. A critical question concerns our capacity to detect and diagnose a new viral infection. The aetiological agent of the COVID‐19 pandemic was defined in record time, but early public health control measures at the place of its origin were not able to prevent the worldwide spread of the infection. During the COVID‐19 pandemic, two further viral epidemics were detected. While the aetiological agent of the 2022 monkeypox epidemic was also quickly identified, it took several months to clarify the cause of the 2022 non‐A to E hepatitis epidemic in children. The existence of this new infectious disease entity was quickly notified, but the identification of the aetiological agent turned out to be difficult. In fact, the cause seems to be a complex mixture of genetic risk, the timing of infections due to an interaction with the ongoing pandemic, and the cooperation between multiple viral agents. Research on causes of the 2022 non‐A to E hepatitis epidemic in children represents new challenges to virologists and thus holds some important learning lessons for our understanding of infectious diseases.

## EPIDEMIOLOGICAL STUDIES

The detection of a new form of viral hepatitis was quick. By the end of March 2022, the Royal Hospital for Children in Glasgow had observed, within a 3‐week period, five cases of severe hepatitis of unknown aetiology. However, Hepatitis virus A to E and other hepatotropic viruses were not detected. Retrospectively, eight additional cases have been reported for Scotland since January 2022. This rate—although still small—was 10‐fold higher than the background rate for this condition in previous years. Five of the 13 cases had a recent SARS‐CoV‐2 infection. Five patients were PCR‐positive for adenovirus (Marsh et al., 2022). By 8 April, the case number had increased to 74 paediatric patients in the UK [Acute hepatitis of unknown aetiology—the United Kingdom of Great Britain and Northern Ireland (who.int)]. The public health response consisted of additional toxicological and virological tests in newly identified cases. By 20 April, the case number had risen to 111 patients in the UK. A week later, 55 cases were also reported in the EU, 12 cases were detected in the US and 12 cases were noted in Israel. Affected children were previously healthy and showed jaundice and markedly increased liver transaminase levels requiring hospitalization. Vomiting, diarrhoea and nausea preceded hepatitis. Most children recovered, but in about 10% of the cases, the disease progressed to acute liver failure necessitating liver transplantation. One child died. No common exposure (food, drugs, toxins) was identified. Only two pairs of cases were epidemiologically linked. The most common pathogens detected in these paediatric patients were adenovirus and SARS‐CoV‐2. Subtyping of adenovirus revealed type 41F adenovirus both in European and US American cases. In a rapid risk assessment from the European Centers for Disease Control (ECDC) on 28 April 2022, the leading hypothesis was that ‘a cofactor affecting young children having an adenovirus infection, which would be mild in normal circumstances, triggers a more severe infection or immune‐mediated liver damage’ [Increase in severe acute hepatitis cases of unknown aetiology in children (europa.eu)]. By 27 May, WHO reported 650 probable cases of this condition in 33 countries, but the majority of such cases still came from the UK (*n* = 222), directly followed by the US (*n* = 216); while Italy, Spain and Japan each reported about 30 cases. In Europe, 75% of the patients were younger than 5 years and 14% needed intensive care (12% of them received a liver transplantation). Diagnostically, 61% tested positive for adenovirus, 12% were PCR‐positive for SARS‐CoV‐2. COVID‐19 vaccination was excluded as a risk factor since 84% of the patients were unvaccinated. WHO assessed only a moderate risk for the spread at global level. Adenovirus was found in 75% of the tested cases in the UK, and subtyping identified mostly adenovirus type 41F. Metagenome sequencing in liver and blood samples revealed adenovirus‐associated virus 2 (AAV2) in a small number of cases [Acute hepatitis of unknown aetiology in children—multi‐country (who.int)]. By 8 July, the WHO reported that the number of cases had increased worldwide to 1010 patients fulfilling the case definition for this condition. Half of all cases occurred in Europe, but the incidence in countries lacking viral diagnostics was considered to be underestimated. Five per cent of patients needed liver transplantation, the case fatality rate was 2%. Time from symptom onset to hospitalization was 4 days. No gender bias was observed. In Europe, 52% of patients were adenovirus‐positive by PCR, but this rate was only 9% in Japan [severe acute hepatitis of unknown aetiology in children—multi‐country (who.int)]. In the UK, the peak incidence of this epidemic occurred in April/May 2022 and since August 2022, the case numbers decreased substantially [investigation into acute hepatitis of unknown aetiology in children in England: technical briefing 4 (publishing.service.gov.uk); Vimalesvaran et al., 2023].

## CLINICAL STUDIES

Two reports from *The New England Journal of Medicine* summarized the experience with this condition. Fifty children were referred to the Liver Unit in Birmingham/UK between January to mid‐April 2022 with hepatitis and 44 met the case definition of a non‐A to E hepatitis. Median age was 4 years, 80% were White and except for three patients who suffered from allergy, constipation or autism, all were previously healthy. Presenting symptoms were jaundice in 93%, vomiting in 54%, diarrhoea in 32% and lethargy in 23% of the cases. Serum bilirubin and aminotransferase levels were strongly elevated. Most children had spontaneous improvement, but 14% progressed to acute liver failure, developed encephalopathy and needed liver transplantation. Liver histology showed hepatocyte damage with inflammation and predominantly CD8 T cells. Of the tested patients, 90% were adenovirus‐positive by PCR in blood samples. Sequencing identified adenovirus type 41F. The adenoviral load was 10‐fold higher in patients with liver transplantation than in cases not needing transplantation. The immunohistology of the explanted livers was, however, negative for adenovirus antigens. In addition, 28% of the patients were positive for SARS‐CoV‐2 in molecular tests. Metagenome analysis of liver and blood samples also revealed AAV2 and herpesviruses (Kelgeri et al., 2022).

Epidemiologists noted since November 2021 a four‐fold increase in the UK community prevalence of adenovirus infection among children under the age of 4 compared to the pre‐pandemic and the height of the pandemic period (Acute hepatitis of unknown aetiology technical briefing 2; publishing.service.gov.uk).

Clinicians from Alabama/USA treated between October 2021 and February 2022, a total of 15 children with acute hepatitis, 9 (60%) had no known aetiological cause. The median age was 3 years, and no shared exposure was identified. Major symptoms were vomiting, diarrhoea, fever and fatigue. Physical examination revealed scleral icterus, hepatomegaly and jaundice. Adenovirus was detected in all 9 patients by blood PCR, and 6 showed Epstein Barr virus (EBV), a herpesvirus, but immunological analysis suggested an EBV infection in the past, not an acute EBV infection. Sequence analysis revealed adenovirus type 41, and 3 sequences were identical with an isolate from the Netherlands from 1981. Liver histology showed neither viral cytopathic effects nor adenovirus antigens. Patients with liver failure showed a 10‐fold higher adenovirus load than patients without liver failure. In 109 control patients from the same time period and the same geographical area, only 9% showed the presence of adenovirus in blood PCR. In 2020/2021 three non‐A to E hepatitis patients from the same region showed no adenovirus in the blood. Therefore, the authors did not want to exclude the possibility that the detection of adenovirus in their small case series was an incidental finding (Gutierrez Sanchez et al., 2022).

Metagenome analysis of 13 cases from the UK revealed human herpes virus 6 (HHV‐6) sequences (investigation into acute hepatitis of unknown aetiology in children in England: technical briefing 4; publishing.service.gov.uk). Clinicians from Birmingham/UK reported on three 6‐ to 11‐month‐old female patients with hepatitis that progressed to liver transplantation. The only virological pre‐transplantation finding was a HHV‐6 infection. After liver transplantation, the patients showed HHV‐6 viremia and graft rejection. All three patients died from graft rejection. Immunohistochemical HHV‐6 detection in their native livers was inconclusive but positive in the allograft livers. The clinicians hypothetically linked the 2022 hepatitis epidemic to the lifting of COVID lock‐down measures, which led to the exposure of young children to common childhood infections such as adenovirus and HHV‐6, from which they were protected by constraints on contacts during the pandemic. The researchers suspected that delayed exposure to a common childhood viral infection caused an abnormal anti‐viral immune response.

In a survey from two German university centres, severe childhood hepatitis without known aetiology has been documented since 2001, but the incidence doubled in 2021/2022. In 57 patients, clinical signs suggested an infection: herpesviruses represented the most frequent diagnosis. HHV‐6 was detected in 6, cytomegalovirus (CMV) in 3, herpes simplex virus (HSV) in 3 and Epstein–Barr virus (EBV) in 3. Adenovirus was detected in only 2 patients (Leiskau et al., 2023).

## MOLECULAR STUDIES

Three reports published in *Nature* provided a wealth of molecular information on non‐A to E hepatitis in children from both the UK (Ho et al., 2023; Morfopoulou et al., 2023) and the US (Servellita et al., 2023). The epidemic in Scotland occurred between March and August 2022. An increase in adenovirus infection shortly preceded the unexplained hepatitis epidemic, while SARS‐CoV‐2 cases had already peaked several months earlier. HHV‐6 infections occurred with low prevalence and displayed a moderate peak in early 2020. These researchers conducted a case–control molecular study. The case group comprised 32 children with unexplained hepatitis hospitalized in 2022 showing the typical symptoms for this disease. The children were negative for common hepatitis viruses (hepatitis A, B, C, E, EBV, CMV, HHV‐6 and 7, HSV). Control groups were constituted of 13 age‐matched healthy children, 12 children with adenovirus infection but normal transaminase levels, 33 explained hepatitis cases and 16 contemporaneous healthy controls. Target‐enriched and metagenome sequencing revealed AAV2 in all investigated non‐A to E hepatitis cases, both in plasma and in liver biopsy samples. AAV2 was also detected in contemporaneous control plasma samples, indicating an ongoing surge of AAV2 infection in children from Scotland, but AAV2 viral load was significantly higher in cases than in controls. Cases likewise showed an increased level of IgM antibodies against AAV2 compared to controls suggesting an ongoing primary infection. Cases demonstrated a higher level of HHV‐6 DNA, but not of HHV‐6 RNA than controls indicating a past latent, but not an acute current infection with this herpesvirus. Adenovirus DNA was not significantly elevated in cases over controls. Patients revealed distinct AAV2 sequences belonging to a single clade that was closely related to AAV2 sequences obtained from France between 2004 and 2015. Longitudinal plasma samples from cases showed a positive correlation between AAV2 viral load and transaminase and bilirubin levels. The researchers demonstrated an association of a specific HLA type with cases: 93% of cases compared to 16% of controls showed at least one copy of the HLA‐DRB1*04:01 allele. Liver biopsies provided further valuable insights: in situ hybridization showed the presence of AAV2 RNA within the nuclei and cytoplasm of hepatocytes and endothelial cells, proving the presence of replicating AAV2. Immune typing on liver sections demonstrated a proliferation of epithelial cells and a dense infiltrate of CD4^+^ and CD8^+^ T cells in the liver. The researchers from this large consortium speculated that the HLA association indicates a genetic disease susceptibility where replication of AAV2 in liver cells induced a maladaptive immune response leading to liver damage (Ho et al., 2023).

Morfopoulou et al. (2023) conducted metagenome sequencing in five explanted livers and in six blood samples from UK cases of non‐A to E hepatitis patients that did not need transplantation. Abundant AAV2 reads were detected in the liver and blood samples; lower levels of HHV‐6 were detected in the liver but not in the blood of cases. Metatranscriptomics revealed AAV2 RNA indicating active AAV2 replication in liver and blood, while HHV‐6 and adenovirus RNA were not detected. Nanopore sequencing demonstrated concatamers of AAV2 genomes proving active DNA replication in liver cells. However, no AAV‐2 proteins could be shown by immunohistology or proteome analysis of liver tissue. From a range of controls comprising healthy children, immunocompromised children and children with human adenovirus infections and children with other forms of hepatitis, AAV2 DNA was also identified albeit with lower prevalence and lower viral load, namely in children with adenovirus infection showing both normal and raised aminotransferase levels. HHV‐6 DNA was also detected in the blood of adenovirus‐infected children. AAV2 genome sequences were obtained from cases and clustered with those isolated from controls. These researchers confirmed the association of non‐A to E hepatitis cases with the HLA‐DRB1*04:01 allele suggesting a genetic disposition. Both the transcriptomic and proteomic data revealed an immune dysregulation associated with an activation of B cells and T cells, neutrophils and macrophages as well as an activation of innate immunity pathways in the livers of affected patients.

Servellita et al. (2023) analysed whole blood samples from 16 hospitalized US children fulfilling the US Centers for Disease Control and Prevention (CDC) criteria for this novel form of hepatitis. Overall, 358 cases were reported in the US, 6% of whom needed liver transplantation and 4% died. The 16 investigated case patients did not need liver transplantation, they were selected to be human adenovirus (HAdV) positive, and 71% of the adenoviruses were typed as HAdV‐41. These cases were compared to 113 controls representing age‐ and time‐matched patients either without hepatitis, with hepatitis of defined aetiology, or with acute gastroenteritis or healthy blood donors. Using metagenomic sequencing and viral enrichment and targeted sequencing, up to 93% of cases showed AAV2 with a viral load that was 13‐fold higher than for HAdV‐41. Among the controls only 4% displayed AAV2. EBV and HHV‐6 were detected in the blood of 79% and 50% of cases, respectively, but in less than 1% of controls. Triple and quadruple infections (HAdV, AAV2, EBV, HHV‐6) were detected in the majority of the cases, but rarely in controls who included patients with hepatitis of known aetiology. Viral loads for AAV2 were consistently higher than those for HAdV, EBV, and HHV‐6. The AAV2 genomes from the US cases could be molecularly distinguished but formed a defined subcluster of the AAV2 phylogenetic tree. Thirty‐five substitutions were located in the viral genomes and most were shared with those observed in AAV2 genomes from hepatitis cases in the UK. Substitutions were clustered in the hypervariable region of the capsid VP1 protein of AAV2, which has been suggested to be involved in receptor recognition, virion stability, increased replication and evasion of neutralizing antibodies. SARS‐CoV‐2 was not identified in the cases. Also the US researchers proposed that the social distancing imposed by the COVID‐19 pandemic control measures, followed by the opening of the societies in 2022, exposed children to viruses to which they had not acquired immunological experience at an earlier age.

All three reports point consistently and convincingly to AAV2 as a possible cause or at least a necessary co‐factor for the 2022 non‐A to E hepatitis epidemic in children. However, some caution in the interpretation of the data is still indicated: all three studies are retrospective, case numbers were small and that of liver samples analysed were even smaller. The presence of AAV2 in liver cells might represent bystanders and not the cause for hepatotoxicity which still needs to be demonstrated, for example by infections of liver organoids. Such an analysis is also of clinical importance because, in case of a cytopathic virus infection, antiviral drugs might be indicated while in case of a misguided immune response, immunosuppressive drugs should be tried in these cases of non‐A to E hepatitis (Tacke, 2023).

## SUPPORTING EVIDENCE: WASTEWATER STUDIES

While some questions about the aetiological role of HAdV‐41 and AAV2 in the 2022 hepatitis epidemic remain, a number of reports provide additional support for an association between these viral infections and the 2022 epidemic. For example, the non‐A to E hepatitis cases in children from Ireland showed a perfect temporal correlation with clinically diagnosed HAdV‐41 infections in the population. Both events occurred between March and July 2022, with few if any cases noted in 2020/2021. In addition, wastewater sequencing showed, exactly in this time window, a sharp increase in the detection of both HAdV and AAV2, which were not detected before this epidemic. Viral loads for AAV2 reached in wastewater that of crAss_2 phage, the most abundant human stool virus. The phage DNA showed however no temporal change. SARS‐CoV‐2 showed two peaks: one in October/November 2021 and another in July 2022 when the novel hepatitis epidemic ended in Ireland (Martin et al., 2023). A similar analysis was reported for wastewater from London. Peaks of HAdV‐41 metagenomics reads were observed in February and March 2022; the March peak was accompanied by an AAV2 peak of reads in wastewater. At this time the early non‐A to E hepatitis cases were observed; the epidemic peaked in April 2022 when also a broad peak of SARS‐CoV‐2 infections was observed following the relaxation of the distancing restrictions on 24 February 2022 in England. In clinical samples, HAdV and AAV2 detection was significantly correlated and peak AAV2 detection was seen in 2‐year‐old children (Gates et al., 2023).

## SUPPORTING EVIDENCE: HELPER VIRUS RELATIONSHIPS

The co‐occurrence of AAV2 with HAdV and human herpesviruses EBV and HHV‐6 is easily explained by the nature of AAV2. AAV2 belongs to the Dependoparvovirus genus within the Parvoviridae family. These are small, non‐enveloped viruses of 25 nm in diameter with a single‐stranded DNA (ssDNA) genome of 4.7 kb in length. Adenovirus‐associated viruses (AAV) come in 12 serotypes, which vary in their tissue tropism. After receptor recognition, AAV enters the cell, is transported in the trans‐Golgi network and imported into the nucleus. In the nucleus AAV associates with the nucleolus where it enters a latent state, mostly as a circular episomal DNA. Upon coinfection with a helper virus, AAV enters then a lytic stage. With the support of helper virus proteins, AAV achieves its genome replication and packaging. Adenoviruses, herpesviruses or papillomaviruses can serve as helper viruses (Meier et al., 2020). The intimate relationship with adenoviruses is underlined by the fact that AAV was originally isolated as a contaminant of an adenovirus. It is noteworthy that HHV‐6 is also a helper virus able to support AAV2 DNA replication (Thomson et al., 1991, 1994). Even the higher viral copy number of AAV2 over HAdV and HH‐6 in non‐A to E hepatitis patients, which is consistently reported in clinical studies, can be explained by the biology of AAV. AAV suppresses the transcription of the helper viruses in a way to maintain some necessary expression of helper proteins but to avoid resource competition by an unhampered replication of the helper virus (Meier et al., 2020).

## HEPATOTOXICITY OF THE ASSOCIATED VIRUSES

The common hepatotropic viruses are a group of diverse pathogens which comprise a Picornavirus (Hepatitis A virus, HAV), a Hepdnavirus (Hepatitis B virus, HBV), a Flavivirus (Hepatitis C virus, HCV), a Deltavirus (Hepatitis D virus, HDV) and a Calicivirus (Hepatitis E virus, HEV). More than 500,000 cases of acute viral hepatitis are diagnosed in the US every year; 32% are caused by HAV, 43% by HBV and 21% by HCV. About 4% of hepatitis infections remain unexplained. Many occur after transfusion and in organ transplant recipients. Hepatitis G virus (HGV) and transfusion‐transmitted virus (TTV) have been discussed as additional hepatotropic viruses but their role as genuine human viruses is not established (Curry & Chopra, 2005). Viral hepatitis is also a major health problem in children from both developing and developed countries. Major aetiological agents of infectious paediatric hepatitis are again the five above‐mentioned viruses HAV to HEV (except in the newborn period where bacterial agents such as *E. coli*, *Listeria* and of syphilis dominate). However, a long list of systemic infections can also include hepatitis as a disease expression. In alphabetic and not ranking order these are adenovirus, arbovirus, coxsackievirus, enterovirus, EBV, HSV, HIV, paramyxovirus, Rubella and Varicella zoster virus (Jensen & Balistreri, 2016).

What is known about liver infections caused by the viruses which were discussed to be associated with the 2022 non‐A to E hepatitis epidemic in children? Since this epidemic occurred during the COVID‐19 pandemic, when many children were exposed to SARS‐CoV‐2 and new SARS‐CoV‐2 infection‐associated diseases such as the Multisystem Inflammatory Syndrome in Children (MIS‐C) were described, an analysis has to start with the possible role of SARS‐CoV‐2 infections in the novel hepatitis epidemic.

### SARS‐CoV‐2

In addition to the major respiratory manifestations of a SARS‐CoV‐2 infection, many COVID‐19 patients showed gastrointestinal and even hepatic symptoms. In the early phase of the COVID‐19 pandemic, Chinese researchers reported that 2% to 11% of COVID‐19 patients had liver comorbidities and 14% to 53% of cases showed abnormally elevated aminotransferase levels during disease progression. Liver dysfunction was also associated with severe COVID‐19 disease (Zhang et al., 2020). Abnormal liver tests in the form of elevated transaminase levels were also found in the majority of hospitalized COVID‐19 patients in the US and were likewise associated with more severe COVID‐19 disease (Hundt et al., 2020). In a retrospective cohort study with 1040 COVID‐19 patients from Hong Kong, 23% showed elevated transaminase levels. Transaminase level elevation was again independently associated with more severe COVID‐19 disease. However, antiviral drug use was correlated with transaminase elevation (Yip et al., 2021). US researchers investigated liver status in 220 children with COVID‐19 and 71 patients with MIS‐C. Elevated transaminase levels were found in 36% and 51% of the two cohorts, respectively. Children with elevated transaminases in both cohorts had a more severe disease course with a greater prevalence of multiorgan dysfunction (Perez et al., 2021). A Canadian study compared the transaminase levels in about 4000 children with a molecularly proven SARS‐CoV‐2 infection with those in 4000 children without SARS‐CoV‐2 detection (9% of them had enterovirus/rhinovirus, 5% respiratory syncytial virus and 3% adenovirus detected). The children were seen at the emergency departments for a respiratory infection. Elevated transaminases were measured in 26% of the COVID‐19 and 22% of the non‐COVID‐19 patients, the difference was not statistically significant (Sumner et al., 2023). Another Canadian study compared the incidence of severe liver injury in 4200 hospitalized COVID‐19 patients over the pandemic. In the pre‐Omicron era 0.3% and in the Omicron era 0.4% of the COVID‐19 patients showed severe liver involvement. A liver biopsy in one adult patient, who was positive for SARS‐CoV‐2 and negative for other hepatotropic viruses, showed immune cell infiltration in both portal and lobular regions. Immunochemistry showed SARS‐CoV‐2 spike protein staining associated with the inflammatory regions in the liver (Swain et al., 2023). Liver involvement in paediatric COVID‐19 is usually mild. Severe hepatitis in paediatric COVID‐19 patients is rare and has been associated with complement dysfunction (Antala et al., 2022).

### HHV‐6

HHV‐6 is one of the common childhood infections causing Roseola infantum where an erythematous rash follows fever subsidence (exanthema subitem). A more common manifestation of HHV‐6 than exanthem subitem is fever without rash, which might cause febrile seizures. Rare neurological manifestations and a mononucleosis‐like syndrome have been associated with HHV‐6 infections. HHV‐6 is a problem in immunocompromised hosts where it complicates bone marrow and solid organ transplantation (Straus, 2005). Among 1653 US children younger than 3 years with acute febrile illnesses, 10% had a primary HHV‐6 infection, as demonstrated by viremia documented by PCR and seroconversion. The mean age of the HHV‐6 patients was 6 to 8 months, shortly after the maternally acquired antibody to HHV‐6 went through a nadir in the infants. Antibody titers rose again in the second half of the first year of life. A positive PCR for HHV‐6 in peripheral blood cells was documented in more than 60% of children older than 10 months. No primary HHV‐6 infection was documented in 580 children with nonfebrile illnesses or 350 healthy controls. Thirteen per cent of the children with primary HHV‐6 infection were hospitalized for seizures. A roseola rash developed in 17% of the primary infections. Reactivation of the infection sometimes with a renewed fever was seen in 16% of patients by antibody increases and in 6% by PCR. The principal complication of primary HHV‐6 infection was seizures. Arthralgia, arthritis and encephalopathy were occasionally observed (Hall et al., 1994). HHV‐6 infections associated with hepatitis are very rare in immunocompetent children but have been documented in a few case studies for three neonates (Mendel et al., 1995; Tajiri et al., 1990; Warner et al., 2023) and two infants and a toddler (Asano et al., 1990; Olson et al., 2019; Sobue et al., 1991; Tajiri et al., 1997).

Following a primary infection, HHV‐6 can establish a latent infection and viral DNA can be detected in a variety of tissues (salivary gland, mononuclear blood cells, urinary and genital tract, skin) (Di Luca et al., 1996). Ozaki et al. (2001) investigated the presence of HHV‐6 DNA in liver biopsies from 48 children suffering from different liver diseases. HHV‐6 DNA belonging to the B variant (HHV‐6B) was found with comparable high prevalence (82% vs. 73%) in children with normal and elevated transaminase levels. HHV‐6 DNA was associated with the nuclei of hepatocytes proving that this virus can infect liver cells. HHV‐6 DNA was found with comparable frequency in cases of viral hepatitis and non‐infectious liver diseases. These researchers concluded: ‘Since HHV‐6 DNA is ubiquitous and since infection can be latent, the mere detection of viral DNA in tissues gives no information about the association between HHV‐6 and disease’. They further suggested that the presence of HHV‐6‐specific IgM might not indicate a primary infection, but it could also represent a reaction to a reactivation of a latent virus since 5% of healthy control children also showed HHV‐6‐specific IgM.

### Adenovirus 41

In immunocompetent children adenovirus causes upper respiratory disease (serotypes 1, 2, 4–6), pharyngoconjunctival fever (serotypes 3, 7), haemorrhagic cystitis (serotypes 7, 11, 21), diarrhoea (serotypes 2, 3, 5, 40, 41), intussusception (serotypes 1, 2, 4, 5), and meningoencephalitis (serotypes 2, 6, 7, 12). In adults, adenovirus is mostly associated with acute respiratory disease, pneumonia and epidemic keratoconjunctivitis. Adenovirus hepatitis was not reported in immunocompetent subjects (Baum, 2005). A different pattern was seen in immunosuppressed patients. A large study from Pittsburgh involving 484 paediatric liver transplant recipients (mean age 3 years) documented adenovirus infections in 10% of the recipients. The most common sites of adenoviral infection were the liver (14 patients had definitive adenovirus hepatitis), the lung, and the gastrointestinal tract. Serotypes 1, 2, and 5 were found to be the most frequent adenovirus types; type 5 was commonly associated with hepatitis (Michaels et al., 1992). A study from Seattle documented 51 adenovirus infections in 1051 bone marrow transplantation patients. Their mean age was 19 years and their underlying diseases were leukaemia and aplastic anaemia. Overall, 43 of the adenovirus‐infected patients showed elevated transaminase concentrations; two had definitive adenovirus hepatitis. The most common adenovirus serotype was 5 (39%), followed by serotype 1 (21%) and serotypes 11/34/35 (13%). Coinfection with herpesviruses was frequent: 31 of the 51 adenovirus‐infected patients also showed HSV or CMV infections. The source of the infection seemed to be the reactivation of an endogenous virus (Shields et al., 1985).

### AAV2

Despite substantial work on the development of AAV2 as a vector for liver‐targeted gene therapy, few data have been published on the in vivo biology of AAV2 infection in humans. Seroprevalence studies showed that AAV infections are widespread: about 35% of sera of healthy blood donors from Europe, the US and Australia contained neutralizing antibodies against AAV2, this prevalence was 55% in sera from Africa. Antibodies directed against AAV2 showed the highest titers followed by antibodies against AAV1, AAV7 and AAV8 (Calcedo et al., 2009). In Germany, 30% of healthy subjects younger than 10 years showed IgG serum antibody to AAV2, this prevalence increased to 60% in the 10 to 19 years‐old group. Comparable seroprevalence data were seen in subjects from Brazil and Japan. Females showed a significantly higher seroprevalence; the difference was explained by a hormonal reactivation of latent virus infection since pregnant women showed a particularly high seroprevalence. The investigated sera showed a seroprevalence of 95%, 35% and 55% for IgG antibodies to adenovirus, CMV and HSV, respectively (Erles et al., 1999). When leukocytes from 243 healthy blood donors were investigated for AAV DNA, 34% were positive by PCR. The most prevalent viral type was AAV2, followed by AAV5. In immunosuppressed patients, AAV‐specific DNA could be detected in 76% of the samples, half of them showed mixed infections with different AAV types. Leukocyte separation revealed that AAV resided in CD3 T lymphocytes. In blood donors 7% of the subjects were also positive for HHV‐6 DNA by PCR, this prevalence increased to 14% in immunosuppressed patients (Hüser et al., 2017).

AAV2 is a popular viral vector for gene therapy because it transduces efficiently cultured liver cells and no human pathology has been associated with AAV2 in the past. In a gene therapy trial with 10 haemophilia B patients treated with a liver‐directed AAV vector expressing factor IX to correct the pathology, 8 patients showed moderately increased transaminase levels. A transient peak of transaminase levels occurred at the beginning of the vector treatment (Chowdary et al., 2022) suggesting a residual hepatotoxic activity of AAV vectors. French researchers explored the presence of AAV DNA in frozen liver tissues from 1319 patients with benign and malignant liver tumours. They identified AAV DNA in 18% of non‐tumour liver tissue, half of the positive samples represented AAV2 and half AAV2/AAV13 hybrid viruses. The viral copy number was low and the viral DNA was in episomal form. Transcription of AAV RNA was seen in 64% of the AAV‐positive non‐tumour samples. Transcription was more frequent in young patients suggesting to the authors that in older children a transcriptionally inactive, perhaps not‐episomal viral DNA subsisted after the primary infection. Potential AAV helper viruses were detected in 43% of the liver tissues. HHV‐6 was the most frequently detected helper virus (39%), followed by EBV (6%) and adenovirus (0.5%). AAV DNA was detected in 8% of the tumour tissues with no difference between benign and malignant tumours. Viral episomal forms of AAV were rarely identified in tumours suggesting integration of AAV DNA into the host chromosome. In 30 human hepatocellular carcinoma (HHC), clonal AAV insertions were recurrently identified in six human genes where the AAV insertion triggered oncogenic overexpression challenging the notion of AAV2 as a non‐pathogenic virus (La Bella et al., 2020). Notably, a liver‐specific enhancer‐promoter element was identified in the wild‐type AAV2 genome that is found at the right‐hand inverted terminal repeat of the AAV genome. This 124‐nt sequence which binds several human transcription factors is within the 163‐nt common insertion region of the AAV genome, which has been implicated in the dysregulation of known HCC driver genes (Logan et al., 2017). This gene region is a concern for the safety of AAV2 vectors for gene therapy, but at the same time suggests that AAV2 infections might under appropriate conditions display pathological effects on liver cells.

## LEARNING LESSONS

Alerting the public health system about a novel form of viral hepatitis was quick, as it was for the 2022 monkeypox epidemic in men having sex with men. Clinicians are thus very efficient in warning the public about a new threat after observing even small unusual clusters of infections. Defining an aetiological agent for a novel disease was also quick for the monkeypox epidemic, but it took several months to identify the agent(s) causing this new form of non‐A to E hepatitis. It is a warning that the rapid detection of an aetiological agent as in the case of COVID‐19 or monkeypox cannot be considered as a rule. It might take months to define the aetiology even when the associated viruses are relatively well known, as in the case of the 2022 hepatitis epidemic. The complex task of virologists of defining a causative agent will slow the application of targeted public health measures or effective therapies. The 2022 hepatitis epidemic has confronted virologists with an infectious disease having a multifactorial cause. Polymicrobial infections have been defined in many bacterial infections, the 2022 non‐A to E hepatitis epidemic reminds us that we have to extend this concept to some viral infections. A complex interaction between a specific parvovirus with a specific adenovirus and herpes viruses, most prominently HHV‐6, might be the underlying cause. While none of them is a liver pathogen, under special circumstances, they can develop liver pathology, particularly in the immunocompromised. None on its own seems enough to start a hepatitis epidemic, and additional cofactors are needed to spark an epidemic such as the 2022 hepatitis epidemic in children. The available data suggest that the timing of the virus exposure might play an important role. A delayed and combined exposure to common childhood viral infections might have caused a harmful immune response. The delayed viral exposure has, in the case of the 2022 hepatitis epidemic, been attributed to the social distancing measures imposed during the COVID‐19 pandemic, curtailing the transmission of AAV2, HAdV41, HHV‐6 and EBV as common infections in young children. The lifting of these social distancing measures might have led to an untimely (delayed) and combined virus exposure, instead of an earlier and sequential viral exposure. This hypothesis is in line with current thinking that a dysregulated immune response to a viral infection might cause similar if not greater pathology than the cytotoxic effect of the viral infection itself. A pathology‐enhancing effect of a delayed virus exposure is not without precedence. Delayed exposure to poliovirus due to ameliorated hygiene in industrialized countries led to a more serious pathology in older subjects who were spared poliovirus infections in early childhood. The role of the COVID‐19 pandemic and the timing of its control measures might indeed explain the rapid decline of the unexplained paediatric hepatitis case numbers after August 2022, suggesting that the particular epidemiological conditions for this epidemic were not longer met after summer 2022. A further factor in this complex aetiological network might be a genetic disposition for an immune‐pathological anti‐viral response revealed by the prominence of a specific HLA allele associated with the non‐A to E 2022 hepatitis cases.

While we can greet the ending of the 2022 monkeypox and hepatitis epidemics with a sigh of relief, we should continue with research to understand the reasons for their ending since they might also provide clues to understand factors that can transform a viral epidemic into a viral pandemic.

## AUTHOR CONTRIBUTIONS

**Harald Brüssow:** Conceptualization (lead); writing – original draft (lead).

## CONFLICT OF INTEREST STATEMENT

The author has no conflict of interest.

## FUNDING INFORMATION

The author has not received funding for this article.
